# Mean centering is not necessary in regression analyses, and probably increases the risk of incorrectly interpreting coefficients

**DOI:** 10.3389/fpsyg.2025.1634152

**Published:** 2025-07-16

**Authors:** Lee H. Wurm, Miles Reitan

**Affiliations:** Psychology Department, Gonzaga University, Spokane, WA, United States

**Keywords:** mean centering, multiple regression analysis, statistical software, moderation, hierarchical regression, nonlinear relationships, polynomial regression

## Abstract

Scholars trained in the use of factorial ANOVAs have increasingly begun using linear modelling techniques. When models contain interactions between continuous variables (or powers of them), it has long been argued that it is necessary to mean center prior to conducting the analysis. A review of the recommendations offered in statistical textbooks shows considerable disagreement, with some authors maintaining that centering is necessary, and others arguing that it is more trouble than it is worth. We also find errors in people’s beliefs about how to interpret first-order regression coefficients in moderated regression. These coefficients do not index main effects, whether data have been centered or not, but mischaracterizing them is probably more likely after centering. In this study we review the recommendations, and then provide two demonstrations using ordinary least squares (OLS) regression models with continuous predictors. We show that mean centering has no effect on the numeric estimate, the confidence intervals, or the t- or *p*-values for main effects, interactions, or quadratic terms, provided one knows how to properly assess them. We also highlight some shortcomings of the standardized regression coefficient (β), and note some advantages of the semipartial correlation coefficient (sr). We demonstrate that some aspects of conventional wisdom were probably never correct; other concerns have been removed by advances in computer precision. In OLS models with continuous predictors, mean centering might or might not aid interpretation, but it is not necessary. We close with practical recommendations.

## 1 Introduction

Areas of research that were long characterized by tightly-controlled factorial ANOVAs are seeing an increase in the use of regression-based techniques (van Rij et al., 2020; Wurm and Fisicaro, 2014). This is a positive development, as the tools are far more flexible and powerful than the corresponding ANOVAs, and allow a more natural examination of the roles played by independent or predictor variables. However, this development also brings with it a set of issues relating to the proper use and interpretation of the techniques. Many researchers seem unfamiliar with some of these issues, or perhaps in need of a refresher. Some of this is probably a holdover from people’s training in the realm of ANOVA (Darlington and Hayes, 2017; Hayes et al., 2012; Irwin and McClelland, 2001).

The current study consists of a non-technical discussion plus two demonstrations, and focuses on the issue of mean centering in regression analyses. We address three issues. The main motivation is the contradictory prescriptions given by statistical experts regarding mean centering of continuous variables. The question is most pressing in the case of analyses that contain either an interaction term, as in Equation 1:


(1) $$\hat{Y}=b_{0}+b_{1}X_{1}+b_{2}X_{2}+b_{3}X_{1}X_{2}$$

or a polynomial term, as in Equation 2:


(2) $$\hat{Y}=b_{0}+b_{1}X+b_{2}X^{2}$$

We will explore what mean centering does, and what it does not do, in these contexts.

A related issue addressed in the current paper is the interpretation of first-order regression coefficients in the presence of an interaction term or polynomial term (b_1_ and b_2_ in Equation 1; b_1_ in Equation 2). Here, too, we see conflicting assertions by experts about the proper interpretation of these coefficients. Paradoxically, we think it possible that misinterpretations are *more* likely after centering.

A final issue addressed in this paper will be the use and interpretation of β, the standardized regression coefficient. We will note several problems associated with the calculation and reporting of β, and following Darlington and Hayes (2017) and Warner (2013, 2020), we will point out some advantages of the semipartial correlation coefficient (sr) as a possible alternative.

Our discussion and demonstrations will use ordinary least squares (OLS) regression with continuous predictors. Throughout this paper, we will use b to indicate unstandardized regression coefficients, and β to indicate standardized regression coefficients. For clarity and consistency, when we quote other authors’ work, we will substitute these symbols for those that might have been used in their original text.

## 2 Mean centering

Examination of Equations 1, 2 will make it clear that the first order predictors can be very highly correlated with interaction products or polynomial terms. Mean centering (subtracting the mean from every score) reduces these correlations, sometimes dramatically, and it is this fact that seems to be the foundation for many authors’ views on centering.

Quotations from two seminal books on multiple regression illustrate the thinking: “Very high levels of multicollinearity can lead to technical problems in estimating regression coefficients. Centering variables will often help minimize these problems” (Aiken and West, 1991, pp. 32–33). “The existence of substantial correlation among a set of IV’s creates difficulties usually referred to as ‘the problem of multicollinearity.’ Actually, there are three distinct problems—the substantive interpretation of partial coefficients, their sampling stability, and computational accuracy” (Cohen and Cohen, 1983, p. 115).

Tabachnick and Fidell (1989) were more explicit than most other authors in describing where the trouble comes from: “The problem is that singularity prohibits, and multicollinearity renders unstable, matrix inversion…With multicollinearity, the determinant…is zero to several decimal places. Division by a near-zero determinant produces very large and unstable numbers in the inverted matrix” (p. 87; see also Tabachnick and Fidell, 2001, 2007, 2013). A long list of authors has expressed this same view (Cohen et al., 2003; Fox, 1991; Navarro, 2018; Navarro and Foxcroft, 2022; Navarro et al., 2019; Reinard, 2006).

A related concern was noted by Cohen and Cohen (1983), who said, “Large standard errors mean both a lessened probability of rejecting the null hypothesis and wide confidence intervals” (p. 116; see also Aiken and West, 1991; Bobko, 2001; Fox, 1991).

Many of the preceding claims have been long known to be false (Dalal and Zicker, 2012; Echambadi and Hess, 2007; Hayes et al., 2012), but statistical textbooks contain advice on centering that ranges from describing it as mandatory to concluding that it might not be worth the trouble. Warner (2013) says, “When both predictor are quantitative, is it necessary to center the scores on each predictor before forming the product term that represents the interaction” (p. 632; bold in the original). In a section on the assumptions of regression, Navarro and Foxcroft (2022) list “Uncorrelated predictors,” and then go on to say, “The idea here is that, in a multiple regression model, you do not want your predictors to be too strongly correlated with each other. This is not ‘technically’ an assumption of the regression model, but in practice it’s required” (pp. 317–318; see also Navarro, 2018, and Navarro et al., 2019).

Cohen et al. (2003) do not go so far as to say that centering is required, but they do say, “…*we strongly recommend the centering of all predictors* that enter into higher order interactions in MR prior to analysis” (p. 267; italics in the original). They also noted, though, that “If a predictor has a meaningful zero point, then one may wish to keep the predictor in uncentered form” (p. 266).

Some authors are less enthusiastic about centering. Bobko (2001), for example, says, “…about the only gain from centering is that the new *b*’s will be estimated with less variance” (p. 229). Hayes et al. (2012) go a little further, saying, “…the advantages of [centering] are generally overstated” (p. 9). Darlington (1990) seems to discourage centering, noting that “Measures of unique contribution, such as *b_j_, pr_j_, sr_j_,* or the values of *t* or *F* that test their significance, are affected by centering for all but the *highest*-power term…We usually want statistics that are not affected by centering or similar adjustments “(p. 300; emphasis in the original). Echambadi and Hess (2007) leave no doubt about their conclusion, saying that mean centering “…does not hurt, but it does not help, not one iota” (p. 439), adding, “The cure for collinearity with mean-centering is illusory” (p. 441).

This lack of agreement among experts on the necessity (or not) of centering was the primary motivation for the current study.

### 2.1 Main effects vs. simple/conditional effects

In the context of regression analyses, it is very useful to think of *main effects* as constants. For example, in Equation 3, the slope of the relationship between the dependent variable (DV) and X_1_, controlling for X_2_, is b_1_.


(3) $$\hat{Y}=b_{0}+b_{1}X_{1}+b_{2}X_{2}$$

It is a constant value across all possible values of X_2_.

Interaction means that the effect of a predictor is not a constant; it *depends* on the specific value of one (or more) other predictors. Returning to Equation 1, b_1_ is the slope of the relationship between the DV and X_1_, but only when X_2_ = 0. The slope is not a constant—it is different for every possible value of X_2_. In ANOVA contexts, these specific, non-constant effects are called *simple effects,* and their number is limited by the number of levels of X_2_. In the context of regression analyses, they tend to be called *conditional effects.* With continuous predictors, infinitely many are possible; but the logic is the same as that seen in ANOVA.

### 2.2 What mean centering does

Centering often reduces the intercorrelations between first-order variables and their product or their polynomial terms. This makes researchers feel better about their analyses, but it fails to take into account the difference between essential and non-essential collinearity (e.g., Dalal and Zicker, 2012). *Non-essential collinearity* has to do with how variables are scaled, whereas *essential collinearity* reflects the underlying correlational structure of the predictor set. Mean centering is nothing more than subtracting a constant from each value on a predictor, and as such, it does not affect that variable’s dispersion or its underlying relationship to other predictors in the set. It can only effect non-essential collinearity.

Centering does unquestionably change the values and interpretations of the first-order coefficients (b_1_ and b_2_ in Equation 1; b_1_ in Equation 2). Authors appear to be nearly unanimous in noting that this change makes those coefficients “…likely to be more interpretable” (e.g., Darlington and Hayes, 2017, p. 355). Below, we will explore why this is, and also point out two perhaps unexpected downsides.

### 2.3 What mean centering does not do

Attempts to describe the effects of centering run a very wide range in terms of specificity, clarity, and accuracy. Warner (2013) writes, “The purpose of centering is to reduce the correlation between the product term and the X_1_, X_2_ scores, so that the effects of the X_1_ and X_2_ predictors are distinguishable from the interaction” (p. 632). We will present evidence below that suggests Warner might believe that the coefficients b_1_ and b_2_ in Equation 1 index the main effects of X_1_ and X_2_ (they do not), but either way, it is unclear what it would mean to make such effects “distinguishable” from the interaction.

Reinard (2006) asserts, “Many, but certainly not all, scholars suggest the wisdom of standardizing scores first so that a zero point may be included” (p. 389). This language, too, is problematic. The regression solution is going to “include” a zero point whether scores are standardized or not, though it will have different meanings in the two cases. Weinberg et al. (2023), say that analysts can center “To impart meaning to the b-weights of the first-order-effect variables in an equation that contains an interaction term…” (p. 576). Again, the coefficients in question have meanings, centered or not, and it is the analyst’s responsibility to know what those meanings are.

Some claims made about the effects of centering appear to be wrong. Weinberg et al. (2023) assert that centering increases the power of the statistical test on the interaction in Equation 1, but this is shown to be false by Echambadi and Hess (2007, see also Dalal and Zicker, 2012, Hayes et al., 2012, and Shieh, 2009). As we will see below, centering variables has literally no effect on the value of the regression coefficient b_3_, or its associated standard error, t statistic, or *p* value.

Tabachnick and Fidell (2013, see also Tabachnick and Fidell, 2001, 2007) assert that “Centering an IV… does affect regression coefficients for interactions or powers of IVs…” (p. 158). Centering does not have this effect (e.g., Aiken and West, 1991; Darlington, 1990), which we will demonstrate below.

Tabachnick and Fidell (2013; see also Tabachnick and Fidell, 2001, 2007) appear to be wrong about other things, as well. They say, “Analyses with centered variables lead to the same unstandardized regression coefficients for simple terms in the equation (e.g., b_1_ for X_1_ and b_2_ for X_2_ as when uncentered)” (p. 158). That has long been known to be false (Aiken and West, 1991; Cohen and Cohen, 1983; Cohen et al., 2003), as we will show again below. They go on to say, “The significance test for the interaction also is the same, although the unstandardized regression coefficient is not (e.g., b_3_ for X_1_X_2_)” (pp. 158–159). The first part of this is correct—the significance test for the interaction is the same—but in fact, the unstandardized regression coefficient is, too.

A final thing that centering does not do is convert the first-order regression coefficients (b_1_ and b_2_) to indexes of the main effects of X_1_ and X_2_ in Equation 1. The temptation to interpret them in such a way is strong, especially for researchers experienced with factorial ANOVAs (Darlington and Hayes, 2017; Hayes, 2005; Hayes et al., 2012; Irwin and McClelland, 2001). This temptation is probably made even worse by mean centering. We turn to this topic next.

### 2.4 Interpretation of first-order regression coefficients

In the context of Equation 1, Irwin and McClelland (2001) note, “A common misinterpretation (perhaps as an overgeneralization from ANOVA) is to refer to b_2_ as the ‘main effect’ of X_2_. The term ‘simple effect’ is preferable, because this term refers to the simple relationship between the dependent variable and an independent variable at a particular level of the other independent variable(s)” (p. 102).

Darlington and Hayes (2017) echo this, saying, “We have observed many instances in the literature of investigators interpreting b_1_ and b_2_ as “average” effects or ‘main effects’ as in ANOVA, as the effect of X_1_ and X_2_ collapsing across the other variable. But that is not what b_1_ and b_2_ quantify” (p. 434). They go on to say that despite the fact that statisticians have known this for a long time already, “…the message has been slow to disseminate among users of regression analysis” (p. 434).

To use the language of Darlington (1990), the interaction term X_1_X_2_ in Equation 1 changes the meaning of the first-order terms from “global” to “local:” b_1_ is not a main effect, but a conditional (or simple) effect; it is the effect of X_1_ *at a single specific value of X_2_.* That specific value is zero. By analogy, b_2_ is the conditional effect of X_2_ when X_1_ equals zero. As Irwin and McClelland (2001) put it, “An average or main effect does not change with the addition of a moderator term; the apparent change is the result of a shift from a main effect test to a test of a particular simple effect in the moderator model” (p. 102).

Centering changes the meaning of zero, and here, then, we see the interpretational advantage that might be gained (Aiken and West, 1991; Cohen et al., 2003; see also Darlington, 1990 and Darlington and Hayes, 2017 for the analogous discussion of b_1_ in Equation 2). Without centering, a value of zero on X_1_ or X_2_ might be outside the range of values observed in a study, or even meaningless or impossible. Centering guarantees that b_1_ and b_2_ index effects at values that are at least possible, and probably meaningful.

This does not, though, make them main effects. Main effects are generally thought of as additive, or “general” (Cohen and Cohen, 1983, p. 12) or “constant” (Aiken and West, 1991, p. 38). As Aiken and West (1991) note, “The b_1_ and b_2_ coefficients never represent constant effects of the predictors in the presence of an interaction” (p. 38). The effects indexed by b_1_ and b_2_ in Equation 1 are not constants but variables, whose value depends on the meaning of zero on X_1_ and X_2_.

Irwin and McClelland (2001) point out one reason why the risk of misinterpretation might be heightened here: after mean centering, what’s represented by b_1_ or b_2_ “…is *closer* to what most researchers mean when they refer to the main effect. However, except for special situations (e.g., when the X distribution is exactly symmetric), *it still will not be the same* as the average difference between groups across all levels of X” (p. 102; italics added).

Most models will contain regressors in addition to those involved in an interaction. This makes interpretation of first-order coefficients even more complex, because technically, “b_1_ is the estimated difference in Y between two cases that differ by 1 unit on X_1_ but whose scores on X_2_ equal zero (*and are the same on any covariates*)” (Darlington and Hayes, 2017, p. 386; italics added). In many real datasets, this situation is so statistically unlikely as to render the situation all but impossible.

It should be noted that the foregoing considerations are only relevant if one intends to interpret or test the significance of those first-order coefficients. Darlington (1990) noted that they are unlikely to be of any interest in the context of an interaction. Even if they were main effects—but they are not—Venables (1998) reminds us, “If there is an interaction between factors A and B, it is difficult to see why the main effects for either factor can be of any interest…” (p. 13). Nevertheless, sometimes researchers are interested in such main effects. As we will see below, a hierarchical approach is required in such cases (e.g., Aiken and West, 1991; Bobko, 2001; Cohen and Cohen, 1983).

Finally, it should be highlighted that any interpretational advantage of centering *is not because of reduced collinearity:* Hayes et al. (2012) note that “Although the regression coefficients, t statistics, *p*-values, and measures of effect size are changed for those variables involved in the interaction, this has nothing to do with reducing multicollinearity. These differences are attributable to the effects of rescaling a variable in such a way that the ‘zero’ point is changed” (p. 10).

Readers may be inclined to say that anyone who went to graduate school knows all this, but our recent experiences with colleagues, collaborators, and reviewers of manuscripts and grant proposals suggests that there might be some lingering misapprehension due to some people’s stronger familiarity with factorial ANOVAs. This can even affect how the topics are treated in statistics textbooks, which has obvious and very serious consequences.

An example from Warner’s (2013, 2020) textbook illustrates how difficult it can be to maintain the proper interpretation of these effects. In this example, physical illness symptoms are being predicted by age (X_1_) and number of healthy habits (X_2_), along with their interaction (X_1_X_2_). Both predictors were centered prior to computation of their product (recall from above that Warner believes such centering is necessary). A model was then fitted, using the original, *uncentered* X1 and X2, along with the product of the *centered* versions of those predictors.^1^

Warner (2013) describes the results as follows:

The interaction was statistically significant… Age was also a statistically significant predictor of symptoms (i.e., older persons experienced a higher number of *average* symptoms compared with younger persons). *Overall,* there was a statistically significant decrease in predicted symptoms as number of healthy habits increased. This *main effect* of habits on symptoms should be interpreted in light of the significant interaction between age and habits” (pp. 633–634; italics added).

The words “average,” “overall,” and “main effect” make it clear that Warner believes these to be main effects. They are not.

Warner (2020) includes the same worked example, and the description of the results no longer has the words “overall” or “main.” However, the language continues to suggest that these are being interpreted as main effects, and in a preliminary list of questions used to guide the analysis, Warner asks, “Which of the three predictors (if any) are statistically significant when controlling for other predictors in the model…?” (p. 234). The three predictors are X_1_, X_2_, and the product of their centered versions.

The same problem undercuts the argument of Iacobucci et al. (2016). They examined the effects of mean centering under differing amounts of collinearity, in regression models that included an interaction term. It is clear from their wording that they believe the first-order coefficients to be indexes of main effects: “We ran a regression using the main effects and interaction, X_1_, X_2_, and X_1_X_2_ to predict Y” (p. 1313); “…we focus on the results for the main effect predictors” (p. 1313); “Mean centering…is good practice when testing and reporting on effects of individual predictors” (p. 1314); “…mean centering reduces standard errors and thus benefits *p*-values and the likelihood of finding β_1_ or β_2_ significant” (p. 1313).

Mean centering does not reduce standard errors or benefit the likelihood of a significant result, if one is testing a main effect. What is happening is that a different *conditional* effect is being estimated before vs. after centering. This has been known for a long time, and we will demonstrate it again below.

### 2.5 Review of β

Darlington (1990) notes, “The most widely used measure of a regressor’s relative importance is β, which is defined as the value of b we would find if we standardized all variables including Y to unit variance before performing the regression” (p. 217). The rationale behind the use of β is “…to eliminate the effects of noncomparable raw (original) units” (Cohen and Cohen, 1983, p. 84), and “…to compare the relative impact of each independent variable on the dependent variable” (Sirkin, 2006, p. 530).

Fox and Weisberg (2019) call this practice “dubious” and “misleading” (p. 185). Hayes (2005) had earlier noted some of the problems with β, saying “…some people prefer standardized coefficients because, it is claimed, they allow for comparisons of the relative importance of predictor variables in estimating the outcome, they are comparable across studies that differ in design and the measurement procedures used, and they can be used to compare the effect of one variable on the outcome across subsamples. But none of these claims are unconditionally true” (p. 337).

Darlington and Hayes (2017) suggest that people should not use the term “beta” at all, saying it “is used in so many ways by different people who write about regression that doing so just invites confusion” (p. 48).

What’s more, the apparently straightforward, easy computational procedure is complicated when we have interaction terms (Equation 1) or powers of X (Equation 2). Fox and Weisberg (2019) add, “…it is nonsense to standardize dummy regressors or other contrasts representing a factor, to standardize interaction regressors, or to individually standardize polynomial regressors or regression-spline regressors…” (p. 185). As will be seen, this is precisely what most software does.

Darlington (1990; see also Darlington and Hayes, 2017) notes several conceptual problems with β, while other authors focus on disagreements about how to calculate it in equations with interactions or polynomial terms. Considerations such as this have led some authorities to advise, “Ignore any output referring to standardized regression coefficients” (Tabachnick and Fidell, 2013, p. 159; see also Tabachnick and Fidell, 2001, 2007). Cohen et al. (2003) also say, “The ‘standardized’ solution that accompanies regression analyses containing interactions should be ignored” (p. 283).

These problems have been known about for a very long time, but the field has been reluctant to abandon the measure. We will follow Darlington and Hayes (2017; see also Warner, 2013, 2020) in noting some advantages in using the semipartial correlation coefficient (sr) instead.

### 2.6 Objectives of the current study

The current research had three primary objectives. One was to examine the effects of mean centering on OLS regression analyses with continuous predictors. A second objective was to clarify the interpretation of first-order regression coefficients, in models that have interactions or polynomial terms. The third objective was to discuss the limitations of the standardized regression coefficient (β) as a measure of relationship strength.

Our overarching goals are pedagogical. We use small datasets, with limited information available about the samples; but we are not concerned with the robustness of any particular statistical inference, or its generalizability to new samples. Our pedagogical conclusions can be demonstrated with any existing (or new, randomly-generated) dataset a researcher happens to have on hand.

## 3 Demonstration 1

We examine Equation 1 in the context of a dataset, comparing uncentered with centered results. Even though we agree with the standard cautions (e.g., Darlington, 1990; Venables, 1998), we assume for this demonstration that researchers would be interested not only in the possible interaction between X_1_ and X_2_, but in the separate main effects.

### 3.1 Method

All analyses were conducted in SPSS (IBM Corp, 2023), and confirmed in Jamovi (The Jamovi Project, 2024) and JASP (JASP Team, 2024). Analytic procedures for this demonstration, for these programs and for R (R Core Team, 2024) are available in the Supplementary materials (note that the files there are listed as tables, even though they contain analytic procedures).

For this demonstration, we used the *IceCream* dataset from the “sur” R package (Harel, 2020, GNU General Public License ≥ 2). This dataset consists of 30 cases in which the number of ice cream bars sold was recorded, along with that day’s temperature and relative humidity. In a first analysis, we predicted the number of ice cream bars sold, using as predictors the temperature in degrees Fahrenheit (X_1_), the relative humidity (X_2_), and their product (X_1_X_2_). We then mean-centered temperature and humidity, computed a new product, and repeated the analysis.

Finally, we re-did both analyses using a hierarchical procedure, which is required if one wants to assess the main effects of temperature and of humidity (e.g., Aiken and West, 1991; Bobko, 2001; Cohen and Cohen, 1983).

### 3.2 Results

Table 1 shows the zero-order correlations between interaction term and the individual predictors that were multiplied to create it, before and after centering. As can be seen, mean centering accomplished what many researchers want it to: It dramatically weakened the correlations between the individual predictors and their product. 0.886 and 0.926 have become 0.310 and 0.559. The correlation between temperature and humidity is the same, whether they are centered or not.

**Table 1:** Correlations among predictors before and after mean centering.

Variable	Temp	Humidity	Temp × Humidity
Temp	–	0.649	0.886
Humidity	0.649	–	0.926
Temp × Humidity	0.310	0.559	–

Correlations above the diagonal are for the original variables. Correlations below the diagonal are for the mean-centered variables.

The best-fitting regression equation using the original variables is shown in Equation 4:


(4) $$\begin{array}{c} \hat{Y}=-98.675+(2.968\times \text{Temp})+(2.583\times \text{Humidity})\\ +(-0.027\times \text{Temp}\times \text{Humidity}) \end{array}$$

Equation 5 shows the result using the centered variables:


(5) $$\begin{array}{c} \hat{Y}=165.839+(0.852\times \text{TempC})+(0.542\times \text{HumidityC})\\ +(-0.027\times \text{TempC}\times \text{HumidityC}) \end{array}$$

Centering temperature and humidity led to dramatic changes in b_0_, b_1_, and b_2_. The intercept (b_0_) is of little interest, but if one holds a mistaken belief about what b_1_ and b_2_ index (e.g., Warner, 2013, 2020; Iacobucci et al., 2016), they might erroneously conclude that centering has had a dramatic effect on the main effects. However, as emphasized above, “…care must be taken when interpreting (b_1_ and b_2_) so as to not misinterpret them as if they are ‘main effects’ in an ANOVA sense, or ‘average’ effects, which they very much are not” (Darlington and Hayes, 2017, p. 386).

As expected, centering had no effect on the highest-order effect in the equation—the interaction. In both analyses, it was significant (*p* = 0.016). To visualize it, we defined low and high values on temperature and humidity as one standard deviation below and above the mean, and used those values in the two equations.

**Figure 1:**
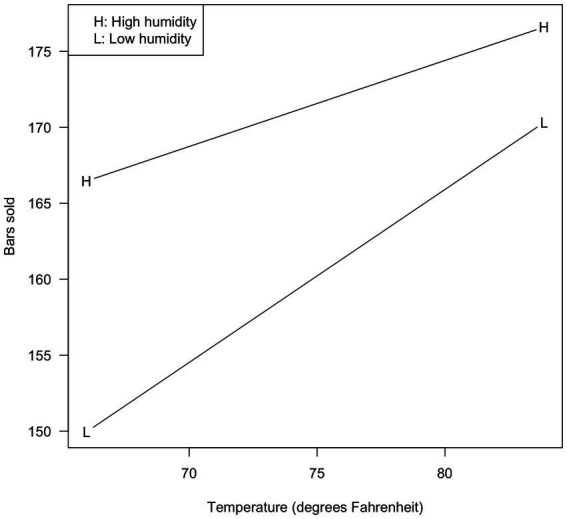
Bars sold as a function of temperature and relative humidity (uncentered variables).

**Figure 2:**
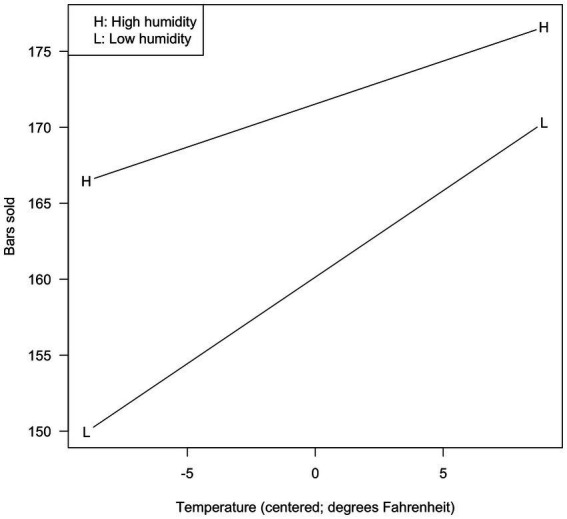
Bars sold as a function of temperature and relative humidity (centered variables).

For temperature, these values were 65.99 and 83.88 (original; −8.94 and 8.94 when centered). For humidity, these values were 67.17 and 88.17 (original; −10.50 and 10.50 when centered). The results are shown in Figure 1 for the original analysis, and Figure 2 for the centered analysis.

As can be seen, the two figures are identical but for the values shown on the X-axis. Higher temperature is associated with more bars sold, and the effect is stronger when the humidity is lower.

Many researchers would consider this sufficient for characterizing the interaction, but some might be interested in the statistical significance of the visualized slopes. Aiken and West (1991) showed that centering never affects the statistical tests on such slopes, so our results are not surprising: The L slope was 1.138 (SE = 0.153, t(26) = 7.419, *p* < 0.001) in both Figures 1, 2; the H slope was 0.566 (SE = 0.167, t(26) = 3.391, *p* = 0.002) in both cases (see Demo1. R in the Supplementary Materials). Nothing was affected by centering.

It is worth noting that any desired conditional effect can be tested; it need not be a standard deviation above or below the mean, which we have used here. This provides yet another piece of evidence that b_1_ and b_2_ are not main effects: If we choose zero as the test value for the slope, we obtain precisely the result that too often gets mistakenly interpreted as a main effect.

Mean centering changes nothing except the labeling of values. In the original data, scores of 0 literally meant 0 degrees Fahrenheit and 0% relative humidity. In the centered data, 0 meant a temperature of 74.93 degrees, and a relative humidity of 77.67%.

For temperature, literal zero is both possible and meaningful, although far from the minimum observed in the dataset. For relative humidity, literal zero may not even be possible. However, such considerations are irrelevant to the regression solution.

Centering does not change what the interaction “looks like” or how we would describe it. Does it change any of the actual statistics? Tables 2, 3 shows the results in more detail, for the original analysis and then the centered analysis. In addition to the unstandardized and standardized regression coefficients, we include the standard error of b, the 95% confidence interval, and sr, the semipartial correlation coefficient. As we noted above, sr has been proposed as an alternative to β (e.g., Darlington, 1990; Darlington and Hayes, 2017). In addition, it has an intuitively appealing interpretation: sr^2^ yields the proportion of variance in the dependent variable uniquely attributable to a predictor (Warner, 2013, 2020).

**Table 2:** Predictors of bars sold (original variables).

Variable	b	SE b	β	sr	95% CI	t	*p*
Constant	−98.675	64.677			[−231.620, 34.269]	−1.526	0.139
Temp (°F)	2.968	0.816	2.229	0.240	[1.290, 4.646]	3.635	0.001
Humidity (%)	2.583	0.851	2.278	0.201	[0.834, 4.333]	3.035	0.005
Temp x Humidity	−0.027	0.011	−3.185	−0.171	[−0.049, −0.006]	−2.586	0.016

Variance inflation factors for Temp, Humidity, and Temp × Humidity were 86.04, 128.88, and 346.93, respectively.

**Table 3:** Predictors of bars sold (centered variables).

Variable	b	SE b	β	sr	95% CI	t	*p*
Constant	165.839	0.992			[163.799, 167.878]	167.146	<0.001
Temp (°F)	0.852	0.116	0.640	0.485	[0.614, 1.091]	7.338	<0.001
Humidity (%)	0.542	0.113	0.478	0.316	[0.309, 0.776]	4.779	<0.001
Temp × Humidity	−0.027	0.011	−0.207	−0.171	[−0.049, −0.006]	−2.586	0.016

Variance inflation factors for Temp, Humidity, and Temp × Humidity were 1.74, 2.29, and 1.47, respectively.

We asserted above that the only effect of interest in these analyses is (probably) the interaction. For that effect, all that differs between the two analyses is the β (−3.185 vs. −0.207). We will have more to say about this in the General Discussion, but both values are wrong (Aiken and West, 1991; Cohen et al., 2003; Friedrich, 1982; Hayes, 2005; Tabachnick and Fidell, 2001, 2007, 2013). We discuss how to obtain the correct value (−0.215) below (see also Demo1. R in the Supplementary materials).

Centering had more noticeable changes on the other lines in the tables, which is to be expected. A reminder of the proper interpretation is warranted here: the b_1_ and b_2_ slopes were dramatically reduced not because the effects of X_1_ and X_2_ got any weaker, but because different conditional effects are being estimated with the centered vs. the original variables. For example, b_1_ is the estimated conditional effect of temperature when humidity equals 0. If that’s literally zero, the slope is 2.968, but if it’s centered 0—in other words, about 77.67%—then the slope is 0.852. Similarly, the slope of the estimated conditional effect of humidity is 2.583 when the temperature is literally 0, or 0.542 when it is about 74.93 degrees Fahrenheit.

As noted in the Introduction, centering ensured that each value represents a conditional effect at a meaningful value on the other variable, but it did not turn these into main effects (Cohen and Cohen, 1983; Irwin and McClelland, 2001). We believe that the statistical significances of b_1_ and b_2_ in either table are of extremely limited interest in most cases (Darlington, 1990; Venables, 1998), and so specific as to render them irrelevant to most research questions. Each of them is a test of one specific slope, conditioned on a single score for the other predictor.

Paradoxically, because the centered conditional effects are closer to the actual main effects, the risk is heightened that they will be misinterpreted as such. If the actual main effects are of genuine interest, they need to be assessed in a different way.

#### 3.2.1 Main effects

Despite the frequent cautions about interpreting main effects in the presence of an interaction, researchers do sometime remain interested in them. What is necessary in this case is a regression model that does not contain the interaction term (Cohen and Cohen, 1983; Bobko, 2001; Irwin and McClelland, 2001). Because of this, our default preference for analyzing this type of data would be the hierarchical analysis we present next. Step 1 of the analysis includes only temperature and humidity, allowing for assessment of their main effects. Step 2 includes temperature, humidity, and their product (see Equation 1), allowing for assessment of the interaction. The simultaneous models presented in Tables 2, 3 are in fact these “Step 2” models.

This was done for the original variables, and then repeated following mean centering. Table 4 shows the results of the hierarchical regression analysis with the original variables. Controlling for humidity, the slope for temperature was 0.878. Controlling for temperature, the slope for humidity was 0.397. These effects can properly be thought of as main effects (or *constant* or *additive* or *general* effects, depending on the author). Any discussion of them would be discouraged by some experts, and must at least be qualified by the significant interaction we already knew about, assessed at Step 2 of this analysis.

**Table 4:** Hierarchical regression predicting bars sold (original variables).

Step	Variable	ΔR^2^	b	SE b	β	sr	95% CI	t	*p*
Step 1		0.857							< 0.001
	Temp		0.878	0.127	0.659	0.501	[0.617, 1.139]	6.894	< 0.001
	Humidity		0.397	0.108	0.350	0.266	[0.174, 0.620]	3.659	0.001
Step 2		0.029							0.016
	T × H		−0.027	0.011	−3.185	−0.171	[−0.049, −0.006]	−2.586	0.016

Table 5 shows the results of the same analysis using the centered predictors. Comparison of Tables 4, 5 show that exactly one value has changed: β for the interaction. We saw both of these (incorrect) values above.

**Table 5:** Hierarchical regression predicting bars sold (centered variables).

Step	Variable	ΔR^2^	b	SE b	β	sr	95% CI	t	*p*
Step 1		0.857							< 0.001
	Temp		0.878	0.127	0.659	0.501	[0.617, 1.139]	6.894	< 0.001
	Humidity		0.397	0.108	0.350	0.266	[0.174, 0.620]	3.659	0.001
Step 2		0.029							0.016
	T x H		−0.027	0.011	−0.207	−0.171	[−0.049, −0.006]	−2.586	0.016

One of the major conclusions of the current study is now clear: For proper assessment of main effects and interactions in moderated regression, the decision to center makes quite literally no difference.

Visualization of the interaction would be the same as above, because it always requires use of the full equation containing X_1_, X_2_, and their product (Bobko, 2001; Cohen and Cohen, 1983). Full equations are not always reported with hierarchical analyses, but we believe they should be, so that readers understand how the plots were made. There is no way to get to the correct, appropriate plots from the hierarchical analysis that assesses both the main effects and the interaction.

### 3.3 Discussion

Mean centering had no effect on the numeric value, statistical significance, nature, or verbal description of either main effect or the interaction. Likewise, although we did not present them, mean centering had no effect on any indices or statistical tests associated with model fit.

Mean centering did affect the values and significance tests of the conditional effects represented by b_1_ and b_2_ in the full equation containing the interaction term. Centering also changed β for the interaction, but this should not be taken as evidence that mean centering is necessary. As noted, both β values are wrong (Aiken and West, 1991; Cohen et al., 2003; Friedrich, 1982; Hayes, 2005; Tabachnick and Fidell, 2001, 2007, 2013), and some authors discourage the use of βs altogether (Darlington, 1990; Darlington and Hayes, 2017). We will have more to say about this in the General Discussion.

Mean centering is always appropriate, provided the analyst knows the correct interpretation of the conditional effects. The decision of whether or not to center variables rests completely on which description/visualization is preferred by the data analyst. Some might prefer Figure 2, in which a score of 0 indicates the mean temperature. Others might prefer Figure 1, in which the interaction is plotted against actual observed temperatures.

#### 3.3.1 How is it that centering has so little effect?

Table 1 clearly shows reduced intercorrelations after centering, which supports most authors’ idea about the purpose of centering. There have been a number of convincing proofs and derivations showing how it can be that this does not affect the results (e.g., Echambadi and Hess, 2007). Dalal and Zicker (2012) summarize, “…because the relationship between an interaction term and the outcome is best indexed by a *partial* regression coefficient, and not a bivariate correlation, substantive conclusions do not change after centering…” (p. 344; italics added).

We remind readers of the distinction between essential and non-essential collinearity. We noted above that subtracting a constant from each value on a predictor cannot change the underlying structural relationship between that predictor and the rest of the set, and thus cannot affect essential collinearity. Pedhazur (1997) wrote that, “…centering X in the case of essential collinearity does not reduce it, though it may mask it…” (p. 306; see also Belsley, 1984). The supposed benefit of reduced collinearity from mean centering is illusory (Echambadi and Hess, 2007; Hayes et al., 2012).

Iacobucci et al. (2016) wished to substitute the terms “micro” and “macro” multicollinearity here, and argued that people who believe centering reduces collinearity and those who believe it does not are both correct. As noted above, though, their argument hinges on a mischaracterization of X_1_ and X_2_ (and b_1_ and b_2_) as having to do with main effects: They even refer to these as “main effect variables” and “main effect terms” (p. 1309), language we have not previously encountered. Those are not main effects.

Re-examination of the conditional effects in Tables 2, 3 shows that centering does indeed produce values that are closer to the main effects shown in Table 4 or Table 5. However, they will still differ, by an amount that depends on the precise distributions of X_1_ and X_2_ values (Irwin and McClelland, 2001). The centered temperature conditional effect is about 3% off from its correct main effect value, while the centered humidity conditional effect is 37% off from its correct value. In neither Table 2 nor Table 3 is the associated statistical test correct as a test of a main effect.

We now turn to a demonstration in which two predictors (X and X^2^) are correlated 0.995 with each other, and show that mean centering is still not necessary.

## 4 Demonstration 2

Data for this demonstration were collected during an exam given in an undergraduate statistics class. As each exam (*N* = 31) was turned in, the exam proctor wrote down how long that student had worked on it, rounded to the nearest minute.^2^ After the usual grading procedure, these times were matched up with the scores earned by each student.

The examination was a midterm exam covering roughly a fourth of the material covered in a required undergraduate statistics course. Nothing was recorded about this sample of participants other than the time the person spent on the exam and the resulting score, so no further demographic information is available. Based on long-running observations of this population, the average age was probably near 20 years. The sample was likely 75–80% women, and almost entirely psychology majors.

As in Demonstration 1, though, our emphasis is not on the statistical inferences made in this analysis or the extent to which they might generalize to other samples of students. We are concerned solely with the effects of centering the time variable.

The motivation for collecting completion times was the observation made over many years that while exams turned in at the very end of an exam period tended to have lower scores, so did those turned in very quickly. This suggests a parabolic relationship between time and score, which we can model with a quadratic term.

When we regress score (Y) on time (X) and a quadratic term for time (X^2^), the resulting equation takes the form shown in Equation 6:


(6) $$\hat{Y}=b_{0}+b_{1}X+b_{2}X^{2}$$

We saw in Demonstration 1 that an X_1_X_2_ interaction term changes the interpretation of the coefficients for X_1_ and X_2_, making them conditional (“local”) effects. Similarly, the presence of a quadratic term in the equation above affects the meaning of b_1_. Without the quadratic term, its correct (and obvious) interpretation would be as an index of the (linear) relationship between X and Y. With the quadratic term in the equation, b_1_ represents the slope of the parabola at X = 0. As with the conditional effects in Demonstration 1, this coefficient and its associated statistical test might or might not even have a meaningful interpretation at all, depending on what X is and how it is scaled. The coefficient for the quadratic term, b_2_, represents the type and amount of curvature in the parabola. Positive values indicate a U-shaped parabola, and negative values indicate an upside-down U shape.

### 4.1 Method

All analyses were conducted in SPSS (IBM Corp, 2023), and confirmed in Jamovi (The Jamovi Project, 2024) and JASP (JASP Team, 2024). For this demonstration we will only present our preferred hierarchical analysis (original and then centered). Data were analyzed hierarchically. Step 1 of the analysis included Minutes, allowing for assessment of the linear relationship between exam score and time. Step 2 included Minutes and Minutes^2^, allowing for assessment of the quadratic relationship. This was done for the original time variable, and then repeated following mean centering.

Analytic procedures for this demonstration, for these programs and for R (R Core Team, 2024) are available as Supplementary materials (note that the files there are listed as tables, even though they contain analytic procedures).

### 4.2 Results

The mean length of time students spent on the exam was 53.61 min (SD = 12.08). The mean score on the exam was 65.45 (SD = 18.41).

Just as interaction terms are often highly correlated with their individual components, a squared variable is often very highly correlated with the original version. For this dataset, the correlation between Minutes and Minutes^2^ was 0.995 before centering, and 0.510 after centering.

Tables 6, 7 show the results of the hierarchical regression analysis with actual minutes and mean-centered minutes, respectively. The only value that differed was the β for the quadratic term, which has gone from −4.136 to −0.472. As in the case of the interaction analyses in Demonstration 1, both of these β values are wrong (Aiken and West, 1991; Cohen et al., 2003; Friedrich, 1982; Hayes, 2005; Tabachnick and Fidell, 2001, 2007, 2013). We discuss how to obtain the correct value (−0.437) below (see also Demo2. R in the Supplementary materials).

**Table 6:** Hierarchical regression predicting exam score (original variables).

Step	Variable	ΔR^2^	b	SE b	β	sr	95% CI	t	*p*
Step 1		0.128							0.048
	Minutes (linear)		−0.545	0.264	−0.358	−0.358	[−1.085, −0.004]	−2.062	0.048
Step 2		0.165							0.016
	Minutes (quadratic)		−0.055	0.022	−4.136	−0.406	[−0.099, −0.011]	−2.557	0.016

Variance inflation figures for Minutes and Minutes^2^ were both 103.56.

**Table 7:** Hierarchical regression predicting exam score (centered variables).

Step	Variable	ΔR^2^	b	SE b	β	sr	95% CI	t	*p*
Step 1		0.128							0.048
	Minutes (linear)		−0.545	0.264	−0.358	−0.358	[−1.085, −0.004]	−2.062	0.048
Step 2		0.165							0.016
	Minutes (quadratic)		−0.055	0.022	−0.472	−0.406	[−0.099, −0.011]	−2.557	0.016

Variance inflation figures for Minutes and Minutes^2^ were both 1.35.

#### 4.2.1 Visualizing the quadratic relationship

The full equation containing both Minutes and Minutes^2^ must be used to visualize the quadratic effect (Bobko, 2001; Cohen and Cohen, 1983). The equation for the original time variable is shown in Equation 7:


(7) $$\hat{Y}=-76.530+(5.727\times \text{Minutes})+(-0.055\times {\text{Minutes}}^{2})$$

Notice that if we erroneously interpreted b_1_ as the linear effect of time, we would conclude that scores go up the longer students take on the exam. In analyses of this type, Cohen and Cohen (1983) note that “…b_1_ in the quadratic equation is, at most, of academic interest; it is certainly *not* a test of whether there is a significant linear component in the regression” (p. 229; italics in the original). Step 1 of the correct hierarchical analysis showed us that the linear slope for time was −0.545, irrespective of whether we used the original or centered variable.

Equation 8 shows the result for the centered variables:


(8) $$\hat{Y}=72.231+(-0.178\times \text{MinutesC})+(-0.055\times {\text{MinutesC}}^{2})$$

**Figure 3:**
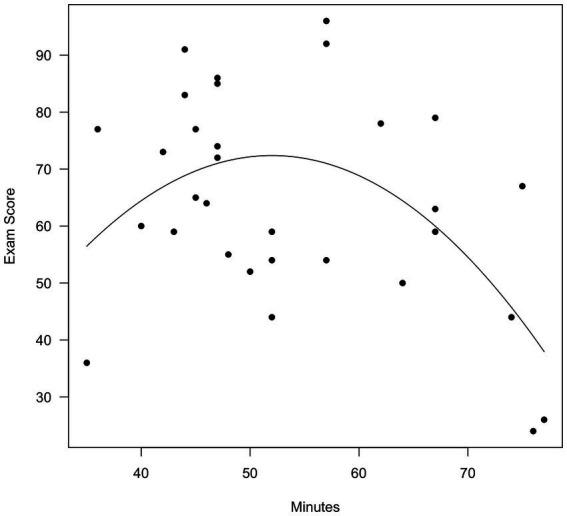
Exam score as a function of time (uncentered).

We defined the X-axis as a vector running from the minimum to the maximum observed value of Minutes. We then plotted the individual data points, and drew the parabola indicated by the Step 2 equation. The resulting plots are shown in Figure 3 (original time) and 4 (centered time).

**Figure 4:**
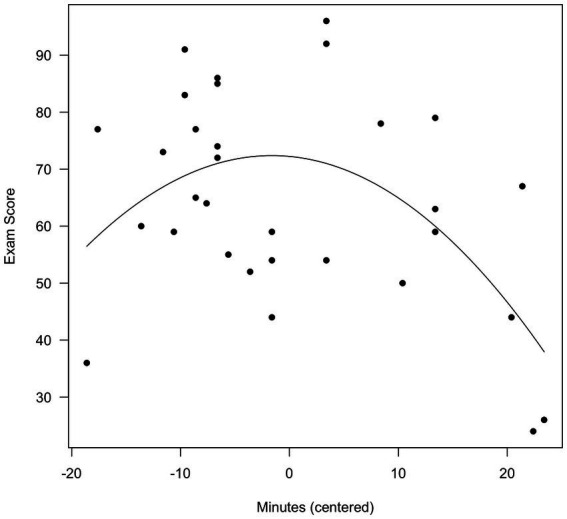
Exam score as a function of time (centered).

As is evident, mean centering made no difference in the nature of the effect. In Figure 3, a score of 0 on Minutes (not shown) means that the student had worked on the exam for 0 min. In Figure 4, a score of 0 Minutes (centered) means that the student had worked on the exam for 53.61 min. Mean centering is always appropriate, and the decision would be made depending on which visualization the analyst prefers.

### 4.3 Discussion

Mean centering affected nothing having to do with the linear “main” effect of time, or the nature of the quadratic relationship, or any of the *p* values or confidence intervals or indices of model fit. It did dramatically change β for the quadratic term, but both values were incorrect, once again highlighting one of the shortcomings of that measure.

Because centering changes the meaning of a score of 0, it changed the conditional effect of time (from 5.727 to −0.178). Neither of these represents the linear effect of time; they represent the conditional (“local”) effect of time, i.e., the slope of the parabola, either at the moment the student is handed the exam (0 = 0 min), or 53.61 min later (0 = the mean completion time). These values are needed for visualizing the quadratic effect, but as above, we maintain that they are almost certainly not of theoretical interest (Darlington, 1990; Venables, 1998).

The conclusion here is the same as in Demonstration 1: Mean centering is not required. Some analysts might find Figure 4 preferable, in which a time of 0 indicates 53.61 min. We find Figure 3 preferable, in which a time of 0 indicates 0 min.

## 5 General discussion

As we have noted in both demonstrations, mean centering is always an appropriate choice for a data analyst to make, provided they know the correct interpretation of the effects being estimated. The current study demonstrates very clearly, though, that centering is not necessary in OLS regression analyses. It might aid interpretation of specific conditional effects—and it might not. It has no effect on the results that are likely to be of most interest to researchers, such as main effects, interactions, and quadratic effects, if the analyses are done correctly.

We are not the first the make these claims or to provide demonstrations. Darlington and Hayes (2017) noted, “Thus, you will often find people describing how they mean-centered X_1_ and X_2_ prior to producing the product ‘to avoid the problems produced by collinearity.’ This myth has been widely debunked…” (p. 435). Echambadi and Hess (2007) provided convincing demonstrations of many of the things we have shown, and concluded, “Whether we estimate the uncentered moderated regression equation or the mean-centered equation, all the point estimates, standard errors and *t* statistics of the main effects, all simple effects, and interaction effects are identical and will be computed with the same accuracy by modern double-precision statistical packages. This is also true of the overall measures of accuracy such as *R^2^* and adjusted-*R^2^*” (p. 444; see also Dalal and Zicker, 2012).

Friedman and Wall (2005) wrote that because of improvements in computational precision, “…multicollinearity does not affect standard errors of regression coefficients in ways previously taught” (p. 127). Indeed, “computational accuracy” was dropped from the list of reasons for centering in Cohen et al. (2003), leaving issues related to interpretation and to sampling stability on the list.^3^ Echambadi and Hess (2007) would argue that interpretation is the only item that properly belongs on any such list of considerations: “…mean-centering does not change the computational precision of parameters, the sampling accuracy of the main effects, simple effects, interaction effects, or the overall model R^2^” (p. 443; see also Dalal and Zicker, 2012).

How could so many people be wrong about this, including the authors of statistical textbooks? One key to the apparent dispute seems to be failing to distinguish essential and non-essential multicollinearity. Correlations that have only to do with scaling are reduced, which can lead researchers to conclude that they have (partially) addressed what they believe to be a serious problem, when in fact they have not (Hayes et al., 2012; see also Belsley, 1984; Pedhazur, 1997).

Cohen et al. (2003), while arguing in favor of centering, implicitly acknowledge that it has no effect on essential collinearity. They wrote, “Centering all predictors has interpretational advantages and eliminates confusing nonessential multicollinearity” (p. 267). We think “eliminates” might overstate the case, and we wonder whether it is useful to potentially mask essential collinearity. We are reminded of a statement by Anderson (1963), who said, “…one may well wonder exactly what it means to ask what the data would be like if they were not what they are” (p. 170).

Another key to the ongoing confusion is a misunderstanding of what is represented by first-order coefficients in an analysis with an interaction or a polynomial term. With *uncentered* data, those coefficients might refer to impossible values, and so it should be crystal clear that they cannot represent main effects. However, subtracting a constant from each score does not magically convert them to main effects, even if the constant happens to be the mean. In fact, a number of authors have pointed out that values other than the mean can be subtracted, if they are interesting or meaningful (Darlington, 1990; Cohen and Cohen, 1983). For example, if a researcher (for some reason) had an interest in the effect of temperature when relative humidity is 85%, he or she could center humidity at 85 rather than its mean (77.67). The resulting analysis would show (and test) the conditional effect of temperature when centered humidity is 0 (meaning that actual humidity is 85). Nobody seems to believe that would be a main effect; so why would it be if the researcher had centered at the mean instead of at 85?

Aiken and West (1991) showed that centering never affects the statistical tests on conditional effects, and we provided yet another demonstration of it in connection with Figures 1, 2. In the present hypothetical example, in which a researcher for some reason has a very specific interest in the conditional effect of temperature when humidity is 85%, the results are identical whether one tests that conditional effect by centering humidity at 85 and then specifying a value of 0; or centering it at its mean and then specifying a value of 7.33; or not centering it at all and then specifying a value of 85.^4^

### 5.1 Reasons to consider abandoning β

Despite its widespread use as a standardized measure of relationship strength, we argued above that the standardized regression coefficient (β) suffers from several shortcomings. We have seen in the current study that centering did affect some of the βs produced by SPSS, Jamovi, and JASP. However, those βs are known to be incorrect for analyses that contain interaction terms or powers of X (Aiken and West, 1991; Cohen et al., 2003; Hayes, 2005; Tabachnick and Fidell, 2001, 2007, 2013). We question the use of a measure that software cannot be trusted to reliably compute.

Cohen et al. (2003) explain that the values are wrong because software packages conduct operations in the wrong order. For interaction analyses, X_1_, X_2_, and their product are turned into z-scores (for quadratic analyses, X and X^2^ are turned into z-scores), and then the analysis is conducted on the z-scores of the DV. As Bobko (2001) noted, the software has no way of knowing that there is anything special about a product or power. The proper order, if a standardized solution is desired, is to convert the individual predictor variables (and the DV) to z-scores, and then recompute the product or quadratic term. The *unstandardized* coefficients from this analysis will be the desired βs.^5^

We have included R code in the Supplementary materials to illustrate how to obtain the correct (and the incorrect) values. However, Sirkin (2006) questioned the utility of standardized regression solutions, saying, “…we normally never use the betas in a prediction equation. We would rarely want to make predictions in standard deviation units” (p. 530). Many others have questioned the wisdom of standardizing dummy variables and factors (e.g., Darlington, 1990; Darlington and Hayes, 2017; Fox and Weisberg, 2019).

### 5.2 The semipartial correlation coefficient: an alternative to β?

If our reason for using β is to compare relationship strengths, the semipartiartial correlation coefficient (sr) might be preferable. Recall that β is intended to convey the predicted amount by which a DV changes, in standard deviation units, as a result of a one standard deviation increase in a predictor, with the value of all other predictors remaining the same. As Darlington and Hayes (2017; see also Darlington, 1990) point out, depending on the correlational structure of a dataset, it can be exceedingly rare (or even impossible) for such cases to exist. The result is that β overstates the importance of predictors, except in the special case of complete independence among predictors (including any interaction terms, polynomial terms, and anything else in the model).

The factor by which β exaggerates a predictor’s importance is the square root of the variance inflation factor (VIF).^6^ Because of this, sr can be thought of as a “corrected” β. sr has the same interpretation as β except that it uses the *conditional* distribution of X (Darlington and Hayes, 2017). In other words, it takes into account the correlational structure of the dataset.

A comparison of sr vs. β in all tables of the current study shows that even after mean centering, β exaggerates each predictor’s importance, except in the Step 1 model of Demonstration 2, where there was literally only one predictor (Minutes) in the model.

There is at least one other advantage of sr: Squaring it yields ΔR^2^, the proportion of variance in the DV that is uniquely explainable by a given predictor. This is an intuitively pleasing way to conceptualize effect size, and it always works for sr. It never works for β, except in the special case of complete independence among all predictors. For these and other reasons, Darlington (1990; Darlington and Hayes, 2017) advocate moving away from reporting β at all. We echo this, along with some other recommendations we hope are useful.

### 5.3 Recommendations for regression models with interactions or polynomials

1. If main effects are of interest, the analysis must be done hierarchically.
2. When plotting a visual representation of an interaction or polynomial regression, it is essential to show the full equation from the final step of the hierarchical analysis, with an indication that this is the equation appropriate for plotting.
3. We very strongly recommend reminding readers that the lower-order effects in this equation are not main effects. *This is no less true with centered data.*
4. We recommend not reporting β values at all. For most purposes, we believe that the semipartial correlation coefficient (sr) is more meaningful, both in its original form and, when squared, as a measure of the variance uniquely accounted for. If editors or reviewers insist on βs, we recommend also including the sr values, along with some explanatory text about the many shortcomings of β.

## 6 Limitations

Our demonstrations used OLS regression models with continuous predictors, but we believe our results should apply to linear models in general (see Hayes et al., 2012; Irwin and McClelland, 2001).^7^ The issue of continuous vs. discrete predictors would not seem to be crucial: Mathematically, the results would necessarily be identical if temperature and relative humidity (or minutes) were discrete variables, rather than continuous ones that just happened to be rounded to the nearest integer. Nevertheless, a limitation of the current work is that our conclusions can technically only be applied to OLS models with continuous predictors. Additional work is required to locate the boundaries beyond which our conclusions do not hold.

## Data Availability

The datasets presented in this study can be found in online repositories. The names of the repository/repositories and accession number(s) can be found at: https://osf.io/4vr9m/?view_only=766f91fe2f91448095ebccb39cec79cc.

## Footnotes

^1^We have not seen anyone else use this strategy. Warner (2020) writes that this strategy “makes interpretation of results easier” (p. 233), even though she had acknowledged earlier (Warner, 2013) that it affects no part of the analysis other than the intercept (b_0_ in Equation 1).

^2^Because time is a continuous variable, it has to be rounded somewhere. We judged the nearest minute sufficient for our purposes.

^3^Paradoxically, their recommendation in favor of centering is even stronger than that given previously by subgroups of the same authors, when computational accuracy was still on the list.

^4^In all three analyses, b_1_ = 0.653, SE = 0.145, t(26) = 4.506, *p* < 0.001 (see Demo1.R in the Supplementary materials).

^5^Any βs offered by the software are still likely to be incorrect for product terms and polynomials, and should again be ignored.

^6^VIF is the reciprocal of tolerance, a measure of independence of each predictor from all other predictors in a set.

^7^Dalal and Zicker (2012) point out that additional considerations apply to moderated structural equation models and multilevel models.
