# Supplementary material for: Mean centering is not necessary in regression analyses, and probably increases the risk of incorrectly interpreting coefficients
Source: Front Psychol. 2025 Jul 16;16:1634152. doi: 10.3389/fpsyg.2025.1634152 (PMC12308356; doi:10.3389/fpsyg.2025.1634152)
Supplement: Supplementary file 1 [file Table_1.DOCX]

SPSS Syntax

* Simultaneous analysis, original variables

DESCRIPTIVES VARIABLES=temp relhumid

/STATISTICS=MEAN STDDEV.

COMPUTE product=temp * relhumid.

EXECUTE.

CORRELATIONS

/VARIABLES=temp relhumid product

/PRINT=TWOTAIL NOSIG FULL

/MISSING=PAIRWISE.

REGRESSION

/MISSING LISTWISE

/STATISTICS COEFF OUTS CI(95) R ANOVA COLLIN TOL ZPP

/CRITERIA=PIN(.05) POUT(.10) TOLERANCE(.0001)

/NOORIGIN

/DEPENDENT barsold

/METHOD=ENTER temp relhumid product.

* Center temp and relhumid, and repeat. We have the means from above.

COMPUTE tempC=temp - 74.933.

EXECUTE.

COMPUTE relhumidC=relhumid - 77.667.

EXECUTE.

COMPUTE productC=tempC * relhumidC.

EXECUTE.

CORRELATIONS

/VARIABLES=tempC relhumidC productC

/PRINT=TWOTAIL NOSIG FULL

/MISSING=PAIRWISE.

REGRESSION

/MISSING LISTWISE

/STATISTICS COEFF OUTS CI(95) R ANOVA COLLIN TOL ZPP

/CRITERIA=PIN(.05) POUT(.10) TOLERANCE(.0001)

/NOORIGIN

/DEPENDENT barsold

/METHOD=ENTER tempC relhumidC productC.

* Hierarchical analyses next

REGRESSION

/MISSING LISTWISE

/STATISTICS COEFF OUTS CI(95) R ANOVA CHANGE ZPP

/CRITERIA=PIN(.05) POUT(.10) TOLERANCE(.0001)

/NOORIGIN

/DEPENDENT barsold

/METHOD=ENTER temp relhumid

/METHOD=ENTER product.

REGRESSION

/MISSING LISTWISE

/STATISTICS COEFF OUTS CI(95) R ANOVA CHANGE ZPP

/CRITERIA=PIN(.05) POUT(.10) TOLERANCE(.0001)

/NOORIGIN

/DEPENDENT barsold

/METHOD=ENTER tempC relhumidC

/METHOD=ENTER productC.
