# Supplementary material for: Mean centering is not necessary in regression analyses, and probably increases the risk of incorrectly interpreting coefficients
Source: Front Psychol. 2025 Jul 16;16:1634152. doi: 10.3389/fpsyg.2025.1634152 (PMC12308356; doi:10.3389/fpsyg.2025.1634152)
Supplement: Supplementary file 2 [file Table_2.DOCX]

jamovi

Simultaneous analyses

--------------

<Variables>, <Edit>

Under "Measure type," ensure that barsold, temp, and relhumid are Continuous (jamovi seems to default to Nominal)

<Data>, <Compute>

Call the new variable "product," and in the formula box, enter "temp * relhumid"

<Analyses>, <Regression>, <Correlation Matrix>

Move temp, relhumid, and product over.

<Analyses>, <Regression>, <Linear Regression>

barsold is the Dependent Variable

temp, relhumid, and product are Covariates

Under Model Coefficients, select the "Confidence interval" and "Standardized estimate" checkboxes

Computing semipartial correlations is jamovi is a little clunky. They have to be done one at a time.

<Analyses>, <Regression>, <Partial Correlation>

Move barsold to Variables (and leave it there!)

Click the "Semipartial" radio button

Move temp to Variables, and relhumid and product to Control Variables.

sr will be the value in the upper right portion of the table.

Then shuffle the positions of temp, relhumid, and product to get the other needed values.

Now center temp and relhumid, and then recompute the product. It *should* work to specify that tempC = temp - MEAN(temp), but it doesn't seem to. So we can get the necessary means under <Data>, <Exploration>

<Data>, <Compute>

Call the new variable "tempC," and in the formula box, enter "temp - 74.933"

<Data>, <Compute>

Call the new variable "relhumidC," and in the formula box, enter "relhumid - 77.667"

<Data>, <Compute>

Call the new variable "productC," and in the formula box, enter "tempC * relhumidC"

Now we repeat the analyses from above, using these centered variables:

<Analyses>, <Regression>, <Correlation Matrix>

Move tempC, relhumidC, and productC over.

<Analyses>, <Regression>, <Linear Regression>

barsold is the Dependent Variable

tempC, relhumidC, and productC are Covariates

Under Model Coefficients, select the "Confidence interval" and "Standardized estimate" checkboxes

Computing semipartial correlations:

<Analyses>, <Regression>, <Partial Correlation>

Move barsold in Variables (and leave it there!)

Click the "Semipartial" radio button

Move tempC to Variables, and relhumidC and productC to Control Variables.

sr will be the value in the upper right portion of the table.

Then shuffle the positions of tempC, relhumidC, and productC to get the other needed values.

--------------

To do the hierarchical analyses, we'll use these same variables but set up the analyses differently.

<Analyses>, <Regression>, <Linear Regression>

barsold is the Dependent Variable

Move temp, relhumid, and product to Covariates.

Under "Model Builder" remove product from Block 1 (if no variables are there, move temp and relhumid over).

Click "Add New Block" and add product to Block 2.

Under Model Coefficients, select the "Confidence interval" and "Standardized estimate" checkboxes

The coefficients table can be toggled back and forth between Models 1 and 2. Model 1 has the main effects. Model 2 has the interaction and the conditional effects.

Semipartial correlation coefficients can be computed as above. For Model 1, don't include product.

To do the analysis with centered variables:

<Analyses>, <Regression>, <Linear Regression>

barsold is the Dependent Variable

Move tempC, relhumidC, and productC to Covariates.

Under "Model Builder" remove productC from Block 1 (if no variables are there, move tempC and relhumidC over).

Click "Add New Block" and add productC to Block 2.

Under Model Coefficients, select the "Confidence interval" and "Standardized estimate" checkboxes

Semipartial correlation coefficients can be computed as above. For Model 1, don't include productC.
