# Supplementary material for: Mean centering is not necessary in regression analyses, and probably increases the risk of incorrectly interpreting coefficients
Source: Front Psychol. 2025 Jul 16;16:1634152. doi: 10.3389/fpsyg.2025.1634152 (PMC12308356; doi:10.3389/fpsyg.2025.1634152)
Supplement: Supplementary file 3 [file Table_3.DOCX]

JASP

Simultaneous analyses

--------------

<Edit Data>, <Insert>, <Insert column after>

Change Name to "product," choose Computed with R code, enter "temp * relhumid," and click Compute column

<Analyses>, <Regression>, <Classical>, <Correlation>

Move temp, relhumid, and product over.

<Analyses>, <Regression>, <Classical>, <Linear Regression>

barsold is the Dependent Variable

temp, relhumid, and product are Covariates

Under Statistics, select the "Confidence interval" and "Part and partial correlations" checkboxes

Now center temp and relhumid, and then recompute the product.

<Edit Data>, <Insert>, <Insert column after>

Change Name to "tempC," choose Computed with R code, enter "temp - mean(temp)," and click Compute column

<Insert>, <Insert column after>

Click down in the spreadsheet or you might unintentionally edit your newest variable

Change name to "relhumidC," choose Computed with R code, enter "relhumid - mean(relhumid)," and click Compute column

<Insert>, <Insert column after>

Click down in the spreadsheet or you might unintentionally edit your newest variable

Change Name to "productC," choose Computed with R code, enter "tempC * relhumidC," and click Compute column

Then repeat the analyses from above, using these centered variables:

<Analyses>, <Regression>, <Classical>, <Correlation>

Move tempC, relhumidC, and productC over.

<Analyses>, <Regression>, <Classical>, <Linear Regression>

barsold is the Dependent Variable

tempC, relhumidC, and productC are Covariates

Under Statistics, select the "Confidence interval" and "Part and partial correlations" checkboxes

--------------

To do the hierarchical analyses, we'll use these same variables but set up the analyses differently.

<Analyses>, <Regression>, <Classical>, <Linear Regression>

barsold is the Dependent Variable

Move temp, relhumid, and product to Covariates.

Under "Model," check the boxes for "Add to null model" for temp and for relhumid.

Under Statistics, select the "Confidence interval" and "Part and partial correlations" checkboxes

The H_0_ model in the JASP output is our Step 1. The H_1_ model is our Step 2.

To do the analysis with centered variables:

<Analyses>, <Regression>, <Classical>, <Linear Regression>

barsold is the Dependent Variable

Move tempC, relhumidC, and productC to Covariates.

Under "Model," check the boxes for "Add to null model" for tempC and for relhumidC.

Under Statistics, select the "Confidence interval" and "Part and partial correlations" checkboxes
