# Supplementary material for: Mean centering is not necessary in regression analyses, and probably increases the risk of incorrectly interpreting coefficients
Source: Front Psychol. 2025 Jul 16;16:1634152. doi: 10.3389/fpsyg.2025.1634152 (PMC12308356; doi:10.3389/fpsyg.2025.1634152)
Supplement: Supplementary file 4 [file Table_4.DOCX]

DESCRIPTIVES VARIABLES=Score Minutes

/STATISTICS=MEAN STDDEV.

COMPUTE MinutesSq=Minutes * Minutes.

EXECUTE.

CORRELATIONS

/VARIABLES=Minutes MinutesSq

/PRINT=TWOTAIL NOSIG FULL

/MISSING=PAIRWISE.

REGRESSION

/MISSING LISTWISE

/STATISTICS COEFF OUTS CI(95) R ANOVA CHANGE COLLIN TOL ZPP

/CRITERIA=PIN(.05) POUT(.10) TOLERANCE(.0001)

/NOORIGIN

/DEPENDENT Score

/METHOD=ENTER Minutes

/METHOD=ENTER MinutesSq.

* Center Minutes and repeat. We have the mean from above.

COMPUTE MinutesC=Minutes - 53.613.

EXECUTE.

COMPUTE MinutesCSq=MinutesC * MinutesC.

EXECUTE.

CORRELATIONS

/VARIABLES=MinutesC MinutesCSq

/PRINT=TWOTAIL NOSIG FULL

/MISSING=PAIRWISE.

REGRESSION

/MISSING LISTWISE

/STATISTICS COEFF OUTS CI(95) R ANOVA CHANGE COLLIN TOL ZPP

/CRITERIA=PIN(.05) POUT(.10) TOLERANCE(.0001)

/NOORIGIN

/DEPENDENT Score

/METHOD=ENTER MinutesC

/METHOD=ENTER MinutesCSq.
