# Supplementary material for: Mean centering is not necessary in regression analyses, and probably increases the risk of incorrectly interpreting coefficients
Source: Front Psychol. 2025 Jul 16;16:1634152. doi: 10.3389/fpsyg.2025.1634152 (PMC12308356; doi:10.3389/fpsyg.2025.1634152)
Supplement: Supplementary file 5 [file Table_5.DOCX]

jamovi

<Variables>, <Edit>

Under "Measure type," ensure that Score and Minutes are Continuous (jamovi seems to default to Nominal)

<Data>, <Compute>

Call the new variable "MinutesSq," and in the formula box, enter "Minutes * Minutes"

<Analyses>, <Regression>, <Correlation Matrix>

Move Minutes and MinutesSq over.

<Analyses>, <Regression>, <Linear Regression>

Score is the Dependent Variable

Move Minutes and MinutesSq to Covariates

Under "Model Builder" remove MinutesSq from Block 1 (if no variables are there, move Minutes over).

Click "Add New Block" and add MinutesSq to Block 2.

Under Model Coefficients, select the "Confidence interval" and "Standardized estimate" checkboxes

The coefficients table can be toggled back and forth between Models 1 and 2.

Computing semipartial correlations is jamovi is a little clunky. They have to be done one at a time. And in this example, because Model 1 has one and only one predictor, the sr value will come out just the same as the zero-order correlation between Score and Minutes. But here's how to get it:

<Analyses>, <Regression>, <Partial Correlation>

Move Score to Variables (and leave it there!)

Click the "Semipartial" radio button

Move Minutes to Variables. We have no Control Variables at Step 1, so this value is it.

For Model 2, move Minutes out of Variables and put it into Control Variables. Move MinutesSq to Variables. The sr value will be in the upper right of the table.

Now center Minutes and then recompute the quadratic term. It *should* work to specify that MinutesC = Minutes - MEAN(Minutes), but it doesn't seem to. So we can get the necessary means under <Data>, <Exploration>

<Data>, <Compute>

Call the new variable "MinutesC," and in the formula box, enter "Minutes - 53.613"

<Data>, <Compute>

Call the new variable "MinutesCSq," and in the formula box, enter "MinutesC * MinutesC"

Then repeat the analyses from above, using these centered variables:

<Analyses>, <Regression>, <Correlation Matrix>

Move MinutesC and MinutesCSq over.

<Analyses>, <Regression>, <Linear Regression>

Score is the Dependent Variable

Move MinutesC and MinutesCSq to Covariates

Under "Model Builder" remove MinutesCSq from Block 1 (if no variables are there, move MinutesC over).

Click "Add New Block" and add MinutesCSq to Block 2.

Under Model Coefficients, select the "Confidence interval" and "Standardized estimate" checkboxes

Computing semipartial correlations, as above:

<Analyses>, <Regression>, <Partial Correlation>

Move Score to Variables (and leave it there!)

Click the "Semipartial" radio button

Move MinutesC to Variables. We have no Control Variables at Step 1, so this value is it.

For Model 2, move MinutesC out of Variables and put it into Control Variables. Move MinutesCSq to Variables. The sr value will be in the upper right of the table.
