# Supplementary material for: Mean centering is not necessary in regression analyses, and probably increases the risk of incorrectly interpreting coefficients
Source: Front Psychol. 2025 Jul 16;16:1634152. doi: 10.3389/fpsyg.2025.1634152 (PMC12308356; doi:10.3389/fpsyg.2025.1634152)
Supplement: Supplementary file 6 [file Table_6.DOCX]

JASP

<Edit Data>, <Insert>, <Insert column after>

Change Name to "MinutesSq," choose Computed with R code, enter "Minutes * Minutes," and click Compute column

<Analyses>, <Regression>, <Classical>, <Correlation>

Move Minutes and MinutesSq over.

<Analyses>, <Regression>, <Classical>, <Linear Regression>

Score is the Dependent Variable

Move Minutes and MinutesSq to Covariates.

Under "Model," check the box for "Add to null model" for Minutes.

Under Statistics, select the "Confidence interval" and "Part and partial correlations" checkboxes

Our Step 1 is the H_0_ model in the JASP output. Our Step 2 is the H_1_ model.

Now center Minutes, and then recompute the quadratic term.

<Edit Data>, <Insert>, <Insert column after>

Change Name to "MinutesC," choose Computed with R code, enter "Minutes - mean(Minutes)," and click Compute column

<Insert>, <Insert column after>

Click down in the spreadsheet or you might unintentionally edit your newest variable

Change Name to "MinutesCSq," choose Computed with R code, enter "MinutesC * MinutesC," and click Compute column

Then repeat the analyses from above, using these centered variables:

<Analyses>, <Regression>, <Classical>, <Correlation>

Move MinutesC and MinutesCSq over.

<Analyses>, <Regression>, <Classical>, <Linear Regression>

Score is the Dependent Variable

Move MinutesC and MinutesCSq to Covariates.

Under "Model," check the box for "Add to null model" for MinutesC.

Under Statistics, select the "Confidence interval" and "Part and partial correlations" checkboxes
