# Supplementary material for: Mean centering is not necessary in regression analyses, and probably increases the risk of incorrectly interpreting coefficients
Source: Front Psychol. 2025 Jul 16;16:1634152. doi: 10.3389/fpsyg.2025.1634152 (PMC12308356; doi:10.3389/fpsyg.2025.1634152)
Supplement: Supplementary file 7 [file Table_7.DOCX]

###################################################################

# We focused on SPSS, jamovi, and JASP for this paper, because one of our

# purposes was to demonstrate that software produces incorrect betas in some

# situations. R does not provide betas at all unless one asks for them, and

# we didn't want to have to intentionally obtain incorrect values. But

# we do so below, to illustrate the problem.

#

# Note that we explicitly compute product terms. That is necessary only for

# the calculation of certain correlations, but it also makes the logic

# of what we are doing very explicit.

#

# Analyses for Demonstration 1.

###################################################################

library(ppcor)

d <- read.csv("IceCream.csv")

#### Simultaneous analysis (original variables) ####

d$product <- d$temp * d$relhumid

round(cor(d[,c(2,4,5)]),3)

simul <- lm(barsold ~ temp * relhumid, data = d)

summary(simul)

round(confint(simul),3)

round(spcor(d[,c(3,2,4,5)])$estimate,3)

# sr values will be in the top row, if we list the DV first

Cool <- mean(d$temp) - sd(d$temp)

Warm <- mean(d$temp) + sd(d$temp)

LowRH <- mean(d$relhumid) - sd(d$relhumid)

HighRH <- mean(d$relhumid) + sd(d$relhumid)

Xaxis <- c(Cool, Warm)

b0 <- simul$coef[1]

b1 <- simul$coef[2]

b2 <- simul$coef[3]

b3 <- simul$coef[4]

LowLine <- b0 + b1*Xaxis + b2*LowRH + b3*Xaxis*LowRH

HighLine <- b0 + b1*Xaxis + b2*HighRH + b3*Xaxis*HighRH

jpeg("Figure 1.jpg", width = 180, height = 180, res = 300, units = "mm")

plot(Xaxis, LowLine, pch = "L", las = 1, ylim = c(150, 177),

ylab = "Bars sold", xlab = "Temperature (degrees Fahrenheit)", type = "b")

lines(Xaxis, HighLine, pch = "H", type = "b")

legend("topleft", c("H: High humidity", "L: Low humidity"))

dev.off()

#### Simultaneous analysis (centered variables) ####

d$tempC <- d$temp - mean(d$temp)

d$relhumidC <- d$relhumid - mean(d$relhumid)

d$productC <- d$tempC * d$relhumidC

round(cor(d[,c(6,7,8)]),3)

simulC <- lm(barsold ~ tempC * relhumidC, data = d)

summary(simulC)

round(confint(simulC),3)

round(spcor(d[,c(3,6:8)])$estimate,3)

# sr values will be in the top row, if we list the DV first

CoolC <- mean(d$tempC) - sd(d$tempC)

WarmC <- mean(d$tempC) + sd(d$tempC)

LowRHC <- mean(d$relhumidC) - sd(d$relhumidC)

HighRHC <- mean(d$relhumidC) + sd(d$relhumidC)

XaxisC <- c(CoolC, WarmC)

b0C <- simulC$coef[1]

b1C <- simulC$coef[2]

b2C <- simulC$coef[3]

b3C <- simulC$coef[4]

LowLineC <- b0C + b1C*XaxisC + b2C*LowRHC + b3C*XaxisC*LowRHC

HighLineC <- b0C + b1C*XaxisC + b2C*HighRHC + b3C*XaxisC*HighRHC

jpeg("Figure 2.jpg", width = 180, height = 180, res = 300, units = "mm")

plot(XaxisC, LowLineC, pch = "L", las = 1, ylim = c(150, 177),

ylab = "Bars sold", xlab = "Temperature (centered; degrees Fahrenheit)", type = "b")

lines(XaxisC, HighLineC, pch = "H", type = "b")

legend("topleft", c("H: High humidity", "L: Low humidity"))

dev.off()

## Testing the significance of the conditional effects in Figure 1

# -- original variables --

library(interactions)

sim_slopes(simul, digits = 3, pred = "temp", modx = "relhumid",

johnson_neyman = FALSE, modx.values = "plus-minus")

# The next several lines do the same thing "by hand:"

LowRH <- mean(d$relhumid) - sd(d$relhumid) # we already did this, above

# Center relhumid at this "Low" value

d$testRH <- d$relhumid - LowRH

# Then we re-run our analysis, using this new centered variable, but

# the *only* result of interest is the conditional effect of temp.

summary(lm(barsold ~ temp * testRH, data = d))

# Coefficients:

# Estimate Std. Error t value Pr(>|t|)

# (Intercept) 74.84529 11.03308 6.784 3.37e-07 ***

# temp 1.13834 0.15343 7.419 7.05e-08 *** <-- Our effect

# testRH 2.58340 0.85116 3.035 0.0054 **

# temp:testRH -0.02724 0.01053 -2.586 0.0157 *

# Then we repeat that process for the "High" line in Figure 1.

HighRH <- mean(d$relhumid) + sd(d$relhumid) # we already did this, above

d$testRH <- d$relhumid - HighRH

summary(lm(barsold ~ temp * testRH, data = d))

# Coefficients:

# Estimate Std. Error t value Pr(>|t|)

# (Intercept) 129.09169 13.90309 9.285 9.71e-10 ***

# temp 0.56639 0.16703 3.391 0.00223 ** <-- Our effect

# testRH 2.58340 0.85116 3.035 0.00540 **

# temp:testRH -0.02724 0.01053 -2.586 0.01566 *

# We get identical results for the conditional effects in Figure 2

# -- i.e., with the centered variables

sim_slopes(simulC, digits = 3, pred = "tempC", modx = "relhumidC",

johnson_neyman = FALSE, modx.values = "plus-minus")

# And by hand:

LowRHC <- -sd(d$relhumid)

d$testRHC <- d$relhumidC - LowRHC

summary(lm(barsold ~ tempC * testRHC, data = d))

# Coefficients:

# Estimate Std. Error t value Pr(>|t|)

# (Intercept) 160.14464 1.29283 123.871 < 2e-16 ***

# tempC 1.13834 0.15343 7.419 7.05e-08 *** <-- Our effect

# testRHC 0.54236 0.11349 4.779 6.02e-05 ***

# tempC:testRHC -0.02724 0.01053 -2.586 0.0157 *

HighRHC <- sd(d$relhumid)

d$testRHC <- d$relhumidC - HighRHC

summary(lm(barsold ~ tempC * testRHC, data = d))

# Coefficients:

# Estimate Std. Error t value Pr(>|t|)

# (Intercept) 171.53310 1.77116 96.848 < 2e-16 ***

# tempC 0.56639 0.16703 3.391 0.00223 ** <-- our effect

# testRHC 0.54236 0.11349 4.779 6.02e-05 ***

# tempC:testRHC -0.02724 0.01053 -2.586 0.01566 *

# How to obtain INCORRECT betas in R (these are the betas produced by

# SPSS, jamovi, and JASP, and included in the manuscript).

# Uncentered variables:

z <- as.data.frame(scale(d[,2:5]))

summary(lm(barsold ~ temp + relhumid + product, data = z))

# Centered variables:

zC <- as.data.frame(scale(d[,c(3,6:8)]))

summary(lm(barsold ~ tempC + relhumidC + productC, data = zC))

# How to obtain CORRECT betas in R. It makes no difference whether we

# use the original or the centered variables, because the product gets

# computed after scale().

# Uncentered variables:

z <- as.data.frame(scale(d[,2:4]))

z$product <- z$temp * z$relhumid

summary(lm(barsold ~ temp + relhumid + product, data = z))

# Centered variables:

zC <- as.data.frame(scale(d[,c(3,6:7)]))

zC$productC <- zC$tempC * zC$relhumidC

summary(lm(barsold ~ tempC + relhumidC + productC, data = zC))

#### Hierarchical analysis (original variables) ####

Step1.lm <- lm(barsold ~ temp + relhumid, data = d)

summary(Step1.lm)

round(confint(Step1.lm),3)

round(spcor(d[,c(3,2,4)])$estimate,3)

# sr values will be in the top row, if we list the DV first

Step2.lm <- lm(barsold ~ temp * relhumid, data = d)

summary(Step2.lm)

summary(Step2.lm)$r.squared - summary(Step1.lm)$r.squared

round(confint(Step2.lm),3)

round(spcor(d[,c(3,2,4,5)])$estimate,3)

# sr value(s) will be in the top row, if we list the DV first

#### Hierarchical analysis (centered variables) ####

Step1.lm <- lm(barsold ~ tempC + relhumidC, data = d)

summary(Step1.lm)

round(confint(Step1.lm),3)

round(spcor(d[,c(3,6,7)])$estimate,3)

# sr values will be in the top row, if we list the DV first

Step2.lm <- lm(barsold ~ tempC * relhumidC, data = d)

summary(Step2.lm)$r.squared - summary(Step1.lm)$r.squared

summary(Step2.lm)

round(confint(Step2.lm),3)

round(spcor(d[,c(3,6:8)])$estimate,3)

# sr value(s) will be in the top row, if we list the DV first

## From the General Discussion

# Testing an arbitrary level of humidity (85%), using three equivalent ways.

# Use our original variables, and specify RH = 85

sim_slopes(simul, digits = 3, pred = "temp", modx = "relhumid",

johnson_neyman = FALSE, modx.values = 85)

# Use the centered model; our desired RH is 7.333% above the mean

sim_slopes(simulC, digits = 3, pred = "tempC", modx = "relhumidC",

johnson_neyman = FALSE, modx.values = 7.333)

# By-hand method, centering at 85

d$testRH <- d$relhumid - 85

summary(lm(barsold ~ temp * testRH, data = d))

# Coefficients:

# Estimate Std. Error t value Pr(>|t|)

# (Intercept) 120.91340 11.89992 10.161 1.52e-10 ***

# temp 0.65262 0.14484 4.506 0.000124 *** <-- Our effect

# testRH 2.58340 0.85116 3.035 0.005403 **

# temp:testRH -0.02724 0.01053 -2.586 0.015660 *
