# Supplementary material for: Mean centering is not necessary in regression analyses, and probably increases the risk of incorrectly interpreting coefficients
Source: Front Psychol. 2025 Jul 16;16:1634152. doi: 10.3389/fpsyg.2025.1634152 (PMC12308356; doi:10.3389/fpsyg.2025.1634152)
Supplement: Supplementary file 8 [file Table_8.DOCX]

###################################################################

# We focused on SPSS, jamovi, and JASP for this paper, because one of our

# purposes was to demonstrate that software produces incorrect betas in some

# situations. R does not provide betas at all unless one asks for them, and

# we didn't want to have to intentionally obtain incorrect values. But

# we do so below, to illustrate the problem.

#

# Note that we explicitly compute the quadratic term. That's necessary only

# for the calculation of certain correlations, but it also makes

# the logic of what we are doing very explicit.

#

# Analyses for Demonstration 2.

###################################################################

library(ppcor)

W1 <- read.csv("W1.csv")

#### Hierarchical analysis (original time variable) ####

W1$MinutesSq <- W1$Minutes * W1$Minutes

round(cor(W1[,c(2,4)]),3)

Step1.lm <- lm(Score ~ Minutes, data = W1)

summary(Step1.lm)

round(confint(Step1.lm),3)

round(spcor(W1[,c(3,2)])$estimate,3)

Step2.lm <- lm(Score ~ Minutes + MinutesSq, data = W1)

summary(Step2.lm)$r.squared - summary(Step1.lm)$r.squared

summary(Step2.lm)

round(confint(Step2.lm),3)

round(spcor(W1[,c(3,2,4)])$estimate,3)

# sr value(s) will be in the top row, if we list the DV first

b0 <- summary(Step2.lm)$coef[1]

b1 <- summary(Step2.lm)$coef[2]

b2 <- summary(Step2.lm)$coef[3]

jpeg("Figure 3.jpg", width = 180, height = 180, res = 300, units = "mm")

plot(W1$Minutes, W1$Score, las = 1, pch = 16, xlab = "Minutes", ylab = "Exam Score")

minutes <- seq(min(W1$Minutes), max(W1$Minutes), by = .1)

yhat <- b0 + b1*minutes + b2*minutes*minutes

lines(minutes, yhat)

dev.off()

#### Hierarchical analysis (centered time variable) ####

W1$MinutesC <- W1$Minutes - mean(W1$Minutes)

W1$MinutesCSq <- W1$MinutesC * W1$MinutesC

round(cor(W1[,5:6]),3)

Step1C.lm <- lm(Score ~ MinutesC, data = W1)

summary(Step1C.lm)

round(confint(Step1C.lm),3)

round(spcor(W1[,c(3,5)])$estimate,3)

Step2C.lm <- lm(Score ~ MinutesC + MinutesCSq, data = W1)

summary(Step2C.lm)$r.squared - summary(Step1C.lm)$r.squared

summary(Step2C.lm)

round(confint(Step2C.lm),3)

round(spcor(W1[,c(3,5,6)])$estimate,3)

# sr value(s) will be in the top row, if we list the DV first

b0C <- summary(Step2C.lm)$coef[1]

b1C <- summary(Step2C.lm)$coef[2]

b2C <- summary(Step2C.lm)$coef[3]

jpeg("Figure 4.jpg", width = 180, height = 180, res = 300, units = "mm")

plot(W1$MinutesC, W1$Score, las = 1, pch = 16, xlab = "Minutes (centered)", ylab = "Exam Score")

minutesC <- seq(min(W1$MinutesC), max(W1$MinutesC), by = .1)

yhatC <- b0C + b1C*minutesC + b2C*minutesC*minutesC

lines(minutesC, yhatC)

dev.off()

# How to obtain INCORRECT betas in R (these are the betas produced by

# SPSS, jamovi, and JASP, and included in the manuscript). Note that the

# Step 1 beta is correct. It is the failure to recompute the quadratic term

# after scale() that produces the wrong result.

# Uncentered variables:

z <- as.data.frame(scale(W1[,2:4]))

Step1.lm <- lm(Score ~ Minutes, data = z)

summary(Step1.lm)

Step2.lm <- lm(Score ~ Minutes + MinutesSq, data = z)

summary(Step2.lm)

# Centered variables:

zC <- as.data.frame(scale(W1[,c(3,5:6)]))

Step1C.lm <- lm(Score ~ MinutesC, data = zC)

summary(Step1C.lm)

Step2C.lm <- lm(Score ~ MinutesC + MinutesCSq, data = zC)

summary(Step2C.lm)

# How to obtain CORRECT betas in R. It makes no difference whether we

# use the original or the centered variables, because the quadratic term

# gets computed after scale().

# Uncentered variables:

z <- as.data.frame(scale(W1[,2:3]))

z$MinutesSq <- z$Minutes * z$Minutes

Step1.lm <- lm(Score ~ Minutes, data = z)

summary(Step1.lm)

Step2.lm <- lm(Score ~ Minutes + MinutesSq, data = z)

summary(Step2.lm)

# Centered variables:

zC <- as.data.frame(scale(W1[,c(3,5)]))

zC$MinutesCSq <- zC$MinutesC * zC$MinutesC

Step1C.lm <- lm(Score ~ MinutesC, data = zC)

summary(Step1C.lm)

Step2C.lm <- lm(Score ~ MinutesC + MinutesCSq, data = zC)

summary(Step2C.lm)
